# A selected organosilicone spray adjuvant does not enhance lethal effects of a pyrethroid and carbamate insecticide on honey bees

**DOI:** 10.3389/fphys.2023.1171817

**Published:** 2023-06-01

**Authors:** Anna Wernecke, Jakob H. Eckert, Gabriela Bischoff, Rolf Forster, Jens Pistorius, Richard Odemer

**Affiliations:** ^1^ Julius Kühn-Institut (JKI)—Federal Research Centre for Cultivated Plants, Institute for Bee Protection, Braunschweig, Germany; ^2^ Institute of Microbiology, Technische Universität Braunschweig, Braunschweig, Germany; ^3^ Julius Kühn-Institut (JKI)—Federal Research Centre for Cultivated Plants, Institute for Bee Protection, Berlin, Germany; ^4^ Bundesamt für Verbraucherschutz und Lebensmittelsicherheit (BVL)—Federal Office of Consumer Protection and Food Safety, Braunschweig, Germany

**Keywords:** spray adjuvants, organosilicone surfactants, tank mixtures, honey bee toxicity, contact exposure, insecticides, ecotoxicological risk assessment

## Abstract

As part of the agricultural landscape, non-target organisms, such as bees, may be exposed to a cocktail of agrochemicals including insecticides and spray adjuvants like organosilicone surfactants (OSS). While the risks of insecticides are evaluated extensively in their approval process, in most parts of the world however, authorization of adjuvants is performed without prior examination of the effects on bees. Nevertheless, recent laboratory studies evidence that adjuvants can have a toxicity increasing effect when mixed with insecticides. Therefore, this semi-field study aims to test whether an OSS mixed with insecticides can influence the insecticidal activity causing increased effects on bees and bee colonies under more realistic exposure conditions. To answer this question a pyrethroid (Karate Zeon) and a carbamate (Pirimor Granulat) were applied in a highly bee attractive crop (oil seed rape) during bee flight either alone or mixed with the OSS Break-Thru S 301 at field realistic application rates. The following parameters were assessed: mortality, flower visitation, population and brood development of full-sized bee colonies. Our results show that none of the above mentioned parameters was significantly affected by the insecticides alone or their combination with the adjuvant, except for a reduced flower visitation rate in both carbamate treatments (Tukey-HSD, *p* < 0.05). This indicates that the OSS did not increase mortality to a biologically relevant extent or any of the parameters observed on honey bees and colonies in this trial. Hence, social buffering may have played a crucial role in increasing thresholds for such environmental stressors. We confirm that the results of laboratory studies on individual bees cannot necessarily be extrapolated to the colony level and further trials with additional combinations are required for a well-founded evaluation of these substances.

## 1 Introduction

The global use of plant protection products (PPP) has increased by 37% over the past 30 years to 2,661,124 million tons, with Europe as the third-largest consumer (FAO, 2022). Germany alone accounts for approximately 1,800 registered products that come as herbicide, fungicide, or insecticide formulation (BVL, 2023a). These formulations underwent regulatory risk assessment to guarantee safe use under agricultural field conditions including bee safety (EFSA, 2022). In practice, PPP are often used in tank mixtures with spray adjuvants to enhance physicochemical properties of the spray solution or to increase the efficacy of pesticides (ASTM, 1999).

Adjuvants serve multiple purposes, which include being emulsifiers that ensure products remain well mixed, solvents that help dissolve the active substance ingredient, and surfactants that facilitate penetration of the active substances into foliage (Hazen, 2000). At present (January 2023), 278 adjuvants are approved for field use in Germany (BVL, 2023b), allowing for countless possible mixing combinations with PPP. They are often used as mixing partners in weed control (BVL, 2023b), but can also be combined with insecticides (Ciarlo et al., 2012) and applied to bee-attractive crops (Durant et al., 2021). Unlike PPP, such as insecticides or fungicides, adjuvants do not contain active substances and therefore are subject to less strict testing requirements (EPRS (European Parliamentary Research Service), 2018). Since risk mitigation measures are specific for the PPP or its particular uses in different crops, they do not account for a potential toxicity increase when mixed with adjuvants. As a result, bees may be directly exposed while they forage on flowering crops without toxicological consideration of adjuvant exposure levels (Straw et al., 2022). In few countries, adjuvants are subject to standardized testing requirements in the registration process with non-target organisms such as bees (BVL, 2021).

However, adjuvants and their effects on non-target organisms are poorly understood at present, as ecotoxicological research has focused primarily on active substances and their formulations (Straw et al., 2022). The few existing publications on honey bees (*Apis mellifera*) highlight potential adverse effects of a particular group of adjuvants, which are organosilicone surfactants (OSS) (Ciarlo et al., 2012; Mullin et al., 2016; Fine et al., 2017; Kordecki, 2019; Wernecke et al., 2022). OSS allow extreme spreading and because of their extraordinarily low surface tension they can increase the potential of active substances to enter foliage tissues via stomata (Stevens, 1993) making them a powerful tool to enhance the mode of action of several PPP. Hence, they are widely used worldwide and their market share is steadily increasing (FBI, 2019).

Under laboratory conditions, OSS are known to impair adult bee olfactory learning (Ciarlo et al., 2012), cause brood mortality when used alone and in combination with pathogens (Fine et al., 2017; Kordecki, 2019) and are even considered as stand-alone pesticides (Mullin et al., 2016). Although others (Donovan and Elliot, 2001; Johnson and Percel, 2013; Ricke et al., 2021) and own results (Wernecke et al., 2022) contradict the findings of effects of the adjuvant itself, we found in a laboratory screening a mortality-increasing effect of the OSS Break-Thru S 301 in combination with four out of five insecticide classes when bees were exposed by direct overspray in a spray chamber. Whether these effects can also be observed under field realistic exposure conditions remained unclear.

Out of the four insecticidal active substances showing an increased mortality in the laboratory screening when mixed with the OSS Break-Thru S 301 (Wernecke et al., 2022), pirimicarb (Pirimor Granulat) and lambda-cyhalothrin (Karate Zeon) were identified as mixing partners for further investigation under more realistic exposure conditions due to their frequent use in bee attractive crops such as orchards and oil seed rape in Germany at the time of the study conduct (PAPA, 2022a; PAPA, 2022b).

To evaluate the possible toxicity enhancing properties of the OSS Break-Thru S 301 in combination with these two insecticides we conducted a semi-field study with controlled exposure conditions. Each insecticide and its combination with the adjuvant was tested for effects on mortality, flower visitation, population and brood development. Additional sampling of matrices for subsequent analytical determination of residues allowed to quantify the amount of the active substances in the flowering crop and bees and to investigate the impact of the adjuvant on residues levels and exposure of bees. We hypothesize that the effects on bees by the selected insecticides and combinations with the OSS seen on laboratory level could be reflected in some of the parameters considered in the semi-field study design. In particular, the above-mentioned brood effects are of interest, as they may affect several generations of bees in the long term including over wintering success and not only foragers at the end of their lifespan. The objective of the study was therefore to identify possible risks by tank mixtures of insecticides and OSS for adult honey bees, honey bee brood and honey bee colonies (I), to investigate the influence of adjuvants on residue levels and subsequent exposure of bees (II) and to derive recommendations to enable the protection of bees in agricultural landscapes when exposed to such tank mixtures (III).

## 2 Material and methods

### 2.1 Experimental design and field site

The semi-field study was conducted in May 2021 following OECD Guidance Document No. 75 (OECD, 2007). The trial was planned as a complete block design at a field site in Sickte, near the city of Braunschweig, Germany. Colonies were randomly divided into five groups with each four replicates (Control, Kar, Kar+301, Pir, Pir+301, see Table 1). Four days before application (Days After Treatment minus 4 = DAT-4), each colony was placed in a tunnel tent with a height of 3 m in center and an area of approximately 40 m^2^ of flowering *Brassica napus* (OSR) (BBCH 65). The experimental colonies were relocated to a remote apiary on DAT8 after 7 days of exposure and monitored until DAT20 when the study was terminated. The investigation of all treatments was carried out simultaneously within one trial.

**TABLE 1:** Test substances (TS).

TS	Tradename^a^	Type	Active substance and adjuvant ingredients	Formulation type^b^	Application rate (product/ha)
Kar	Karate Zeon	Insecticide	100 g/L lambda-cyhalothrin	CS	0.075 L
Pir	Pirimor Granulat	Insecticide	500 g/kg pirimicarb	WG	0.75 kg
301	Break-Thru S 301	Adjuvant	1030 g/L polyether-polymethylsiloxane-copolymer (100% w/w)	SL	0.25 L

^a^ Products legally registered in Germany at the time of the trials.

^b^ CS, capsule suspension; SL, soluble concentrate; WG, water dispersible granule.

### 2.2 Honey bees

Twenty healthy and queen-right honey bee colonies (*A. mellifera*) from the institute’s own apiary in Braunschweig, with a hive body of ten combs were used. The colonies were as homogeneous as possible and contained approximately 10,000 bees per colony, provided with at least two honey/nectar and one pollen comb. The queens originated from one breeding line (sisters reared in the test facility in the previous year). No clinical symptoms of adult bee or brood diseases were observed during inspection. All brood stages were present at this time.

### 2.3 Test substances and chemical treatment

Test substances (Table 1) were selected based on our results obtained in a previous laboratory screening study representing candidates with a significant toxicity increase on laboratory level (Wernecke et al., 2022). In total, two insecticides of different classes (lambda-cyhalothrin—pyrethroid; pirimicarb—carbamate) and their combination with an OSS (Break-Thru S 301—superspreader) resulted in four treatment groups. In Germany, both insecticides are classified as not hazardous to bees when used at the maximum recommended application rate or concentration. According to the manufacturer, the tested adjuvant is designed to improve the adhesion of the spray solution to the leaf surface, to ensure maximum wetting, to enhance active substance uptake into the plant and thus to optimize the efficacy of the plant protection measure (Alzchem, 2022).

All treatments were applied during bee flight at 200 L water/ha (DAT0) to flowering OSR with a portable boom sprayer (Schachtner, Munich, Germany). Products were used at their maximum application rate allowed in Germany at the time of the trial (Table 1). The control group was sprayed with tap water. A toxic reference was not used to increase the number of replicates of the treatments. Treatment with OSS alone was not performed since this is not applicable in agricultural practice.

### 2.4 Data collection

#### 2.4.1 Mortality and flower visitation

From DAT-1 until DAT7 bee mortality was assessed daily. To collect and monitor dead bees, each colony was equipped with a modified Gary dead bee trap, which was fixed at the entrance of the hive (Gary, 1960). Dead and moribund worker bees were assessed and summarized to one count. Daily flower visitation from DAT-1 until DAT7 was evaluated using three flight quadrats (1 m^2^) per tunnel, where all foragers were recorded for 1 min each. All assessments were conducted in a randomized order to minimize bias.

#### 2.4.2 Colony development

The total number of bees and brood cells was estimated for each colony using the “Liebefeld method” (Imdorf et al., 1987) to follow the population development of the colonies during the exposure and monitoring phase. The first estimation was performed before application on DAT-3, followed by estimations on DAT3, DAT8, DAT14, and DAT18.

#### 2.4.3 Brood development

On DAT-3 one or two brood combs containing eggs were taken from each replicate of the treatment groups from the center of the brood nest (Brood area Fixing Day = BFD0). Brood development was continuously assessed and monitored until hatching by selecting approximately 600 cells per comb (300/side). Cells on the frame wire were excluded, as well as cells at the edges of the brood nest, since bees do not maintain them equally as the other cells. Images were taken from each comb side with modifications according to Schur et al. (2003). Briefly, the selected combs were uniquely identified on BFD0, and photographs were taken subsequently on four occasions: BFD+6, BFD+11, BFD+17, and BFD+21 (Sony Alpha 7R III with Tamron 70–300 mm at300 mm). Cells were classified and scored according to the scheme in Supplementary Table S1.1 using the software HiveAnalyzer (v. 2.30) (Höferlin et al., 2013). From this evaluation, the brood termination rate (BTR—see Supplementary Method S1 for details) was calculated, to indicate maldevelopment or aborted brood care in the monitored brood cycle.

#### 2.4.4 Sampling and residue analysis

For verification of exposure and investigation of residue behavior of the insecticides alone in comparison to the tank mixture, dead bee samples from dead bee traps and flower samples (10 OSR florescence per tunnel) were collected six different times before and after treatment. Samples were pooled for each treatment group and stored at −20°C until residue analysis.

The target substances were determined and quantified using an analytical multimethod under conditions adapted to the respective sample material (Bischoff et al., 2020). Pirimicarb and its metabolites pirimicarb-desmethyl and pirimicarb-desmethylformamido were identified and quantified by LC-MS/MS and lambda-cyhalothrin by GC-MS/MS.

For LC-MS/MS measurements, a QTRAP 6500 triple stage quadrupole mass spectrometer (SCIEX, Framingham, MA, United States) with electrospray ionization source (ESI, positive mode) coupled to a Nexera X2 HPLC system (SHIMADZU Corp., Kyoto, Japan) was used. For GC-MS/MS measurements, a TSQ 8000 Evo Triple Stage Quadrupole mass spectrometer coupled to a Trace 1,310 gas chromatograph (Thermo Fisher Scientific Inc., Waltham, MA, United States) was used in negative ion chemical ionization (NICI) measurement mode. Target compounds were identified based on their retention time and three characteristic MRM or SRM transitions.

Active substances in the samples were quantified using matrix standards. After 1:100 or 1:1,000 dilution of the extracts, reference standards in solvent were used for quantification due to consequently sufficiently reduced matrix effects. Quantification was performed by the relative peak areas using the internal standard method. Pirimicarb D6 was used for pirmicarb and its metabolites, and fenpropathrin was used as an internal standard for lambda-cyhalothrin.

The analytical method was validated with sample material of bees and oilseed rape (untreated, control samples). The quality parameters of the method (SANTE, 2020) REC (“Recovery”), LOD (“Limit of Detection”), and LOQ (“Limit of Quantification”) relevant for the evaluation of the analytical results are given and explained in the Supplementary Material S2 (Supplementary Tables S2.1, S2.2).

### 2.5 Statistical analysis

A generalized linear mixed model (GLMM) framework to analyze the effects of insecticide exposure on honey bee mortality, flower visitation and colony development was used. Therefore, response variables were No. of dead and moribund bees, No. of foragers, No. of bees and brood cells per replicate, respectively. GLMMs were fit with negative binomial error distribution (to account for overdispersion) and log link.

Treatment, date and their interaction as fixed effects and replicate as random effect were included. To compare groups pairwise, estimated marginal means were calculated and adjusted by the Tukey-HSD method for multiple comparisons for the response variables No. of dead bees and No. of foragers (= adjusted means). Pre-exposure dates were not evaluated. The model outputs can be found in the Supplementary Material.

A one-way ANOVA was performed to compare the BTR in multiple experimental groups, respectively. If applicable, statistically significant results were further tested pairwise with a Student’s t-test. To correct for multiple comparisons, *p*-values were adjusted with the Bonferroni method.

All analyses were performed in R v.4.1.1 (R Core Team, 2021) with the packages glmmTMB v.1.1.2.3 (Brooks et al., 2017), emmeans v.1.7.0 (Lenth, 2021), multcomp v.1.4-17 (Graves et al., 2019), MuMIn v.1.43.17 (Barton, 2020), tidyverse v.1.3.1 (Wickham et al., 2021), and ggpubr v.0.4.0 (Kassambara, 2020). A significance level of *α* = 0.05 was used for all tests, respectively.

## 3 Results

### 3.1 Mortality and flower visitation

**FIGURE 1:**
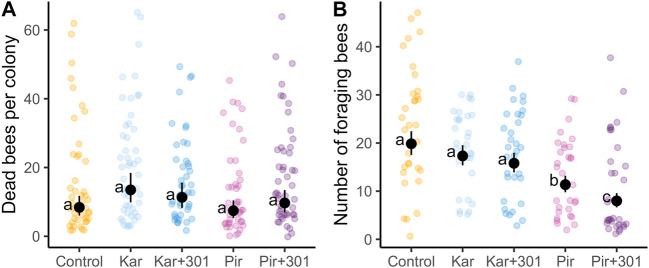
Number of dead bees in dead bee traps and number of foraging bees on exposed plants in the tunnels. Black dots and error bars indicate the adjusted mean (±CL) of dead worker bees in the dead bee trap per colony **(A)** and foragers per quadrat and minute in the tunnels **(B)** of the semi-field trial for all days during the exposure phase (DAT0+1h until DAT7). Treatments are defined as follows: Control (water), Kar (Karate Zeon), Kar+301 (Karate Zeon + Break-Thru S 301), Pir (Pirimor Granulat) and Pir+301 (Pirimor Granulat + Break-Thru S 301) (n_colonies_ = 4). Means that follow the same letter are not significantly different (Tukey-HSD, *p* > 0.05).

After application and during exposure phase in the tunnels (DAT0+1h until DAT7), the overall adjusted mean number of dead bees in the dead bee traps of the treatments was not significantly different from that of the control. Likewise, no statistically significant differences were found between the insecticide applied alone and the related insecticide-adjuvant tank mixture (Tukey-HSD, *p* > 0.05, Figure 1A). The adjusted mean number of dead bees per colony (95% confidence limits) was 8.4 (CL 5.3–10.4) for the control, 13.5 (CL 9.9–18.4) for Kar, 11.3 (CL 8.2–15.6) for Kar+301, 7.4 (CL 5.3–10.4) for Pir, and 9.7 (CL 7.0–13.5) for Pir+301.

During the same period, the adjusted mean flower visitation of the two Karate Zeon treatments was not significantly different from the control. In contrast, it did for the two Pirimor Granulat treatments. Similarly, significantly lower flower visitation was observed for the Pirimor Granulat tank mix treatment compared to Pirimor Granulat alone (Tukey-HSD, *p* < 0.05, Figure 1B). The adjusted mean number of foragers per quadrat (95% confidence limits) was 19.8 (CL 17.5–22.5) for control, 17.3 (CL 15.4–19.6) for Kar, 15.8 (CL 13.9–18.0) for Kar + 301, 11.4 (CL 9.8–13.2) for Pir, and 8.0 (CL 6.6–9.6) for Pir + 301.

**FIGURE 2:**
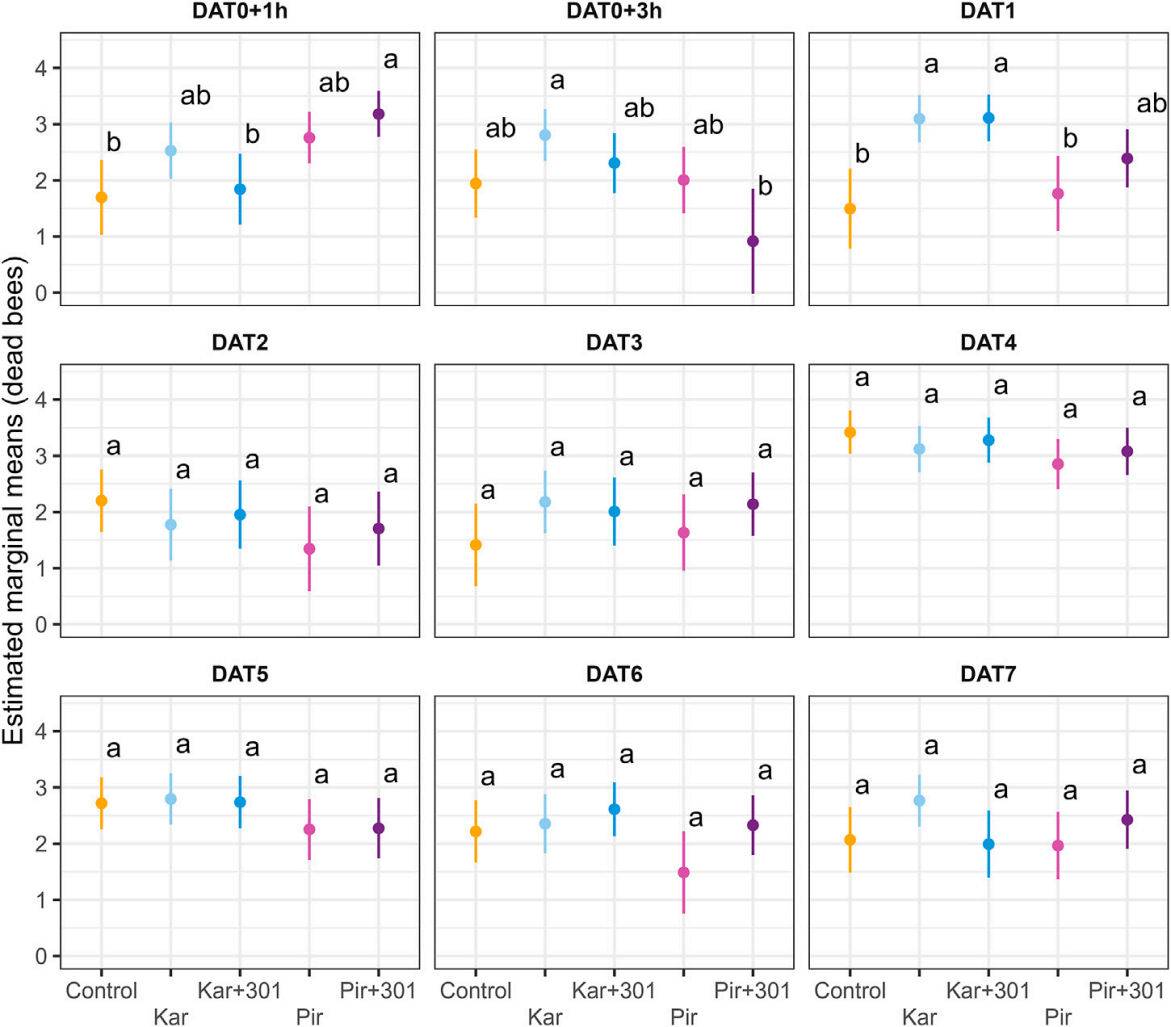
Number of dead bees in dead bee traps on individual days during exposure. Dots and error bars indicate the adjusted mean (±CL) of dead worker bees in the dead bee trap per colony for single days during the exposure phase. Means that follow the same letter are not significantly different (Tukey-HSD, *p* > 0.05) and are reported on the log scale.

**FIGURE 3:**
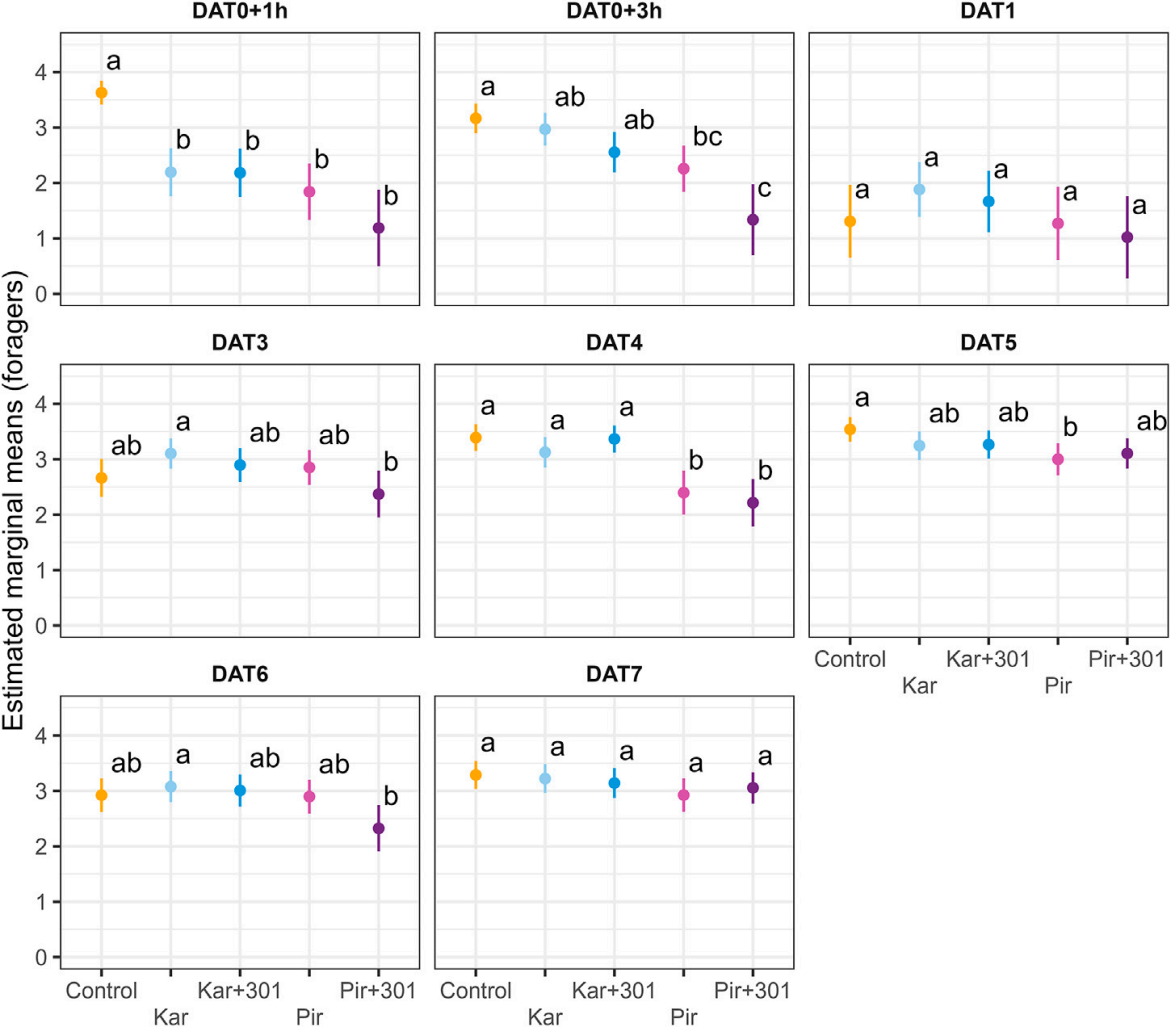
Number of forager bees on exposed plants on individual days during exposure. Dots and error bars indicate the adjusted mean (±CL) number of forager bees per quadrat and minute for single days during the exposure phase. Means that follow the same letter are not significantly different (Tukey-HSD, *p* > 0.05) and are reported on the log scale.

To account for treatment effects on individual dates (interaction term in the model), adjusted means were plotted for dead bees (Figure 2) and for foragers (Figure 3) on the log scale, respectively. The Pir+301 treatment was found to have a significantly higher number of dead bees 1 h after application than in the control and the Kar+301 treatment. However, this leveled off. Compared to the Kar treatment, even significantly fewer dead bees were recorded in the trap 2 h later (Tukey-HSD, *p* < 0.05). On DAT1, significant more bees died in the Kar and Kar+301 treatment than in the control and the Pir treatment. On all other days the treatments were not significantly different from each other. Accordingly, we could not detect any statistically significant differences in mortality induced by the adjuvant up to 7 days after application compared to the insecticide alone.

The mean number of dead bees and foragers per group during the entire tunnel phase (including pre-exposure) is shown in Supplementary Figure S3. In this semi-field trial, the average mortality of the four treatments after application was less than 30 dead worker bees in the trap per colony and time of recording.

Flower visitation was significantly reduced 1 h after application compared to the control (Tukey-HSD, *p* < 0.05). This effect persisted in the two Pirimor treatments until 3 h after treatment and returned on DAT4 (Tukey-HSD, *p* < 0.05). On all other days the treatments were not significantly different from each other. At none of the time points did statistically significant differences occur between tank mix and the individual insecticide. Due to weather conditions, bees were unable to forage on DAT2.

### 3.2 Colony development

**FIGURE 4:**
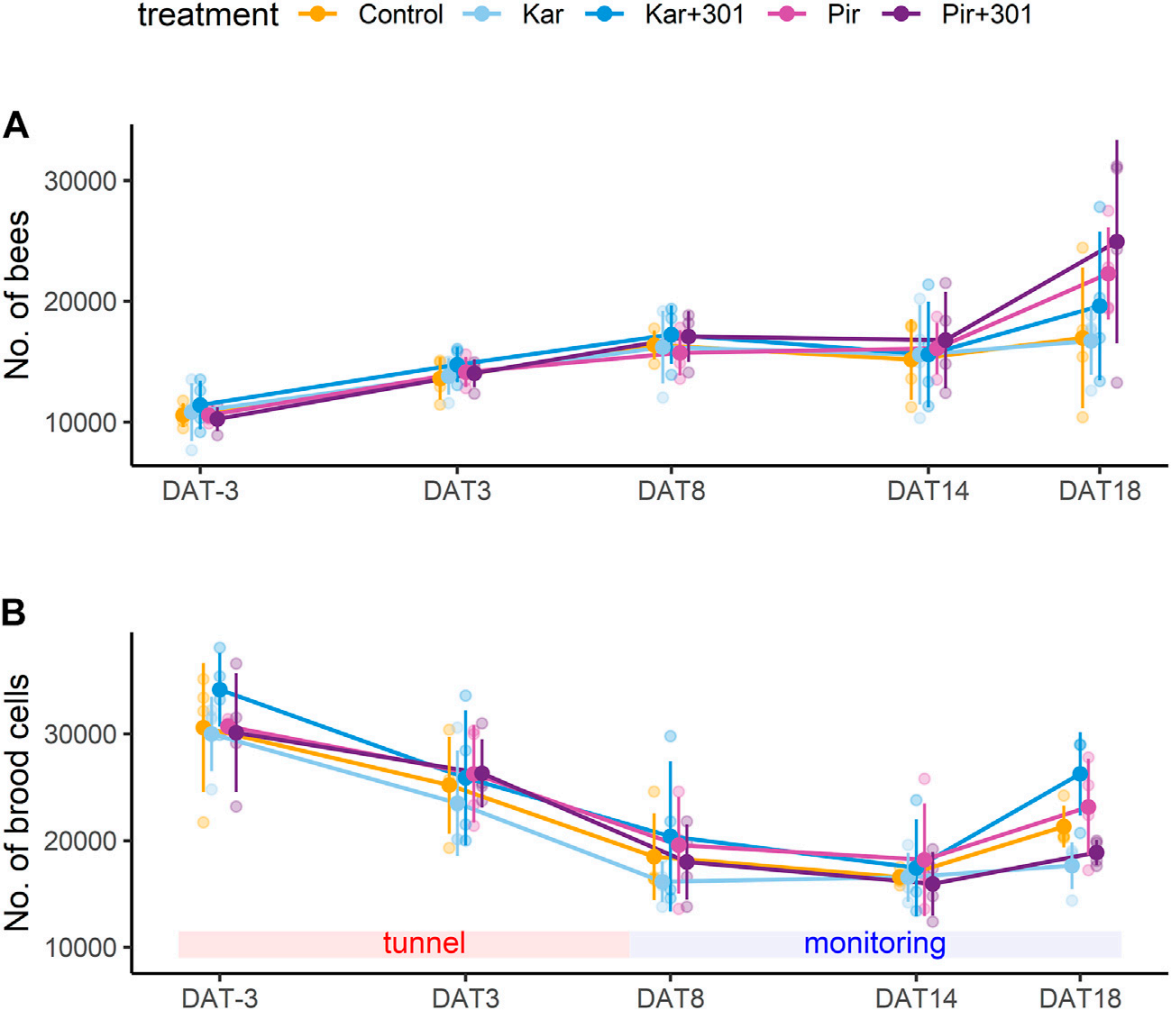
Population dynamics of bees and brood during the experiment. Mean number (±SD) of bees **(A)** and brood cells (sum of eggs, larvae, pupae) **(B)** during the experiment. Both growth parameters were similar in all groups and were not significantly affected by any of the treatments (GLMM, *p* > 0.05).

The randomized assignment of colonies to their respective treatments resulted in initial colony strength differing between groups, although not significantly, with Kar+301 having the most brood cells (Figure 4B). At the time of the evaluations during the tunnel phase and thereafter in the monitoring phase, brood development receded until DAT14, which was followed by a stagnation in the number of bees from DAT8 to DAT14 (Figure 4A). In contrast, 4 days later (DAT18), there was again an increase in brood cells and bees in all treatment groups. Throughout the study, all treatments showed similar numbers of bees and brood development with no significant differences between groups (Figures 4A, B, GLMM, *p* > 0.05). For supplementary informations see Supplementary Material S4.

### 3.3 Brood development and photographic assessment

With reference to the developing time of a worker honey bee from egg to adult (21 to ±1 day; Jay, 1963), we assumed that all eggs should have developed completely at the time of the final brood assessment date (BFD+21). The cumulative number of selected eggs from all replicates was Control (*n* = 2,257), Kar (*n* = 2,400), Kar+301 (*n* = 2,400), Pir (*n* = 2,100), and Pir+301 (*n* = 2,389).

In all groups, successful development was observed of about half of the marked brood cells, Figure 4B.

**FIGURE 5:**
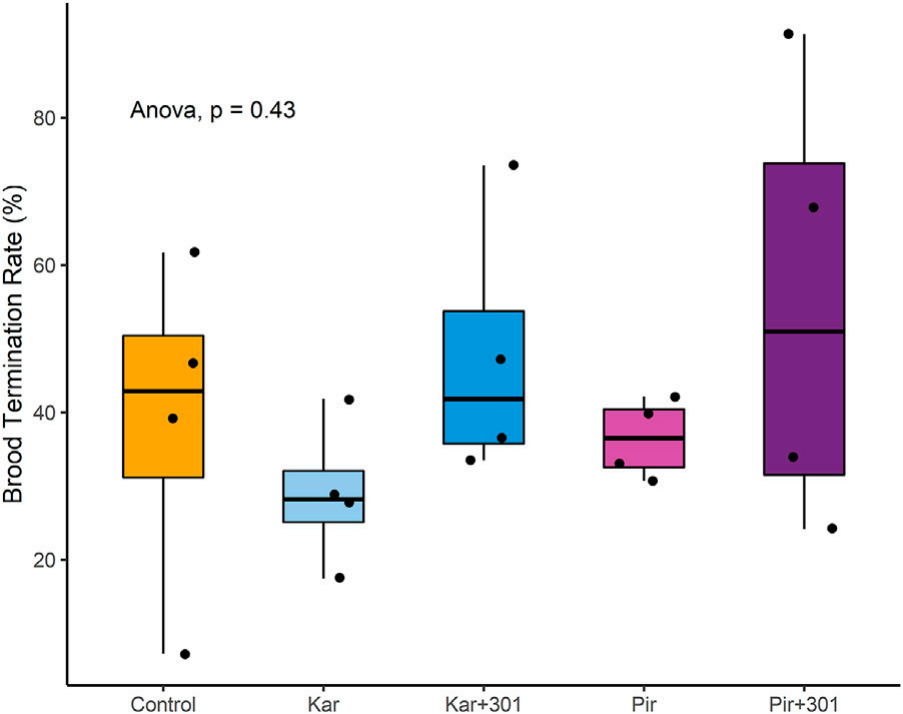
Median brood termination rate (BTR) in percent 18 days after application (BFD+21). The box represents the interquartile range including 50% of the data. The horizontal line within the box indicates the median. The bottom and top of the box indicate the lower (Q1) and upper quartile (Q3). None of the treatments showed significant differences when compared (ANOVA, *p* > 0.05).

On BFD+21, the median termination rate in the groups was Control 42.9%, Kar 28.2%, Kar+301 41.8%, Pir 36.5%, and Pir+301 51.0%, respectively (Figure 5). Due to the large variation within groups, no statistically significant differences were detected (ANOVA, *p* > 0.05).

### 3.4 Residue analysis

Residue analysis of both sampled matrices (dead bees and flowers) confirmed that all colonies in the treated tunnels were exposed to the test substances. On DAT-3, a low pirimicarb content of 0.03 μg/kg was measured in flowers of the pooled sample in the Pir+301 group, but below the LOQ of 0.04 μg/kg established by matrix standards. This is most likely due to contamination during sampling or repackaging of samples in the laboratory.

In addition, in some control tunnels, contamination of the flowers with pirimicarb occurred after application in neighboring tents with values between 0.10 μg/kg and 0.49 μg/kg, which was just above the LOQ. Lambda-cyhalothrin, on the other hand, was not detected in the control during the entire experimental period. The dead bees of the control group showed no contamination with lambda-cyhalothrin or pirimicarb.

**FIGURE 6:**
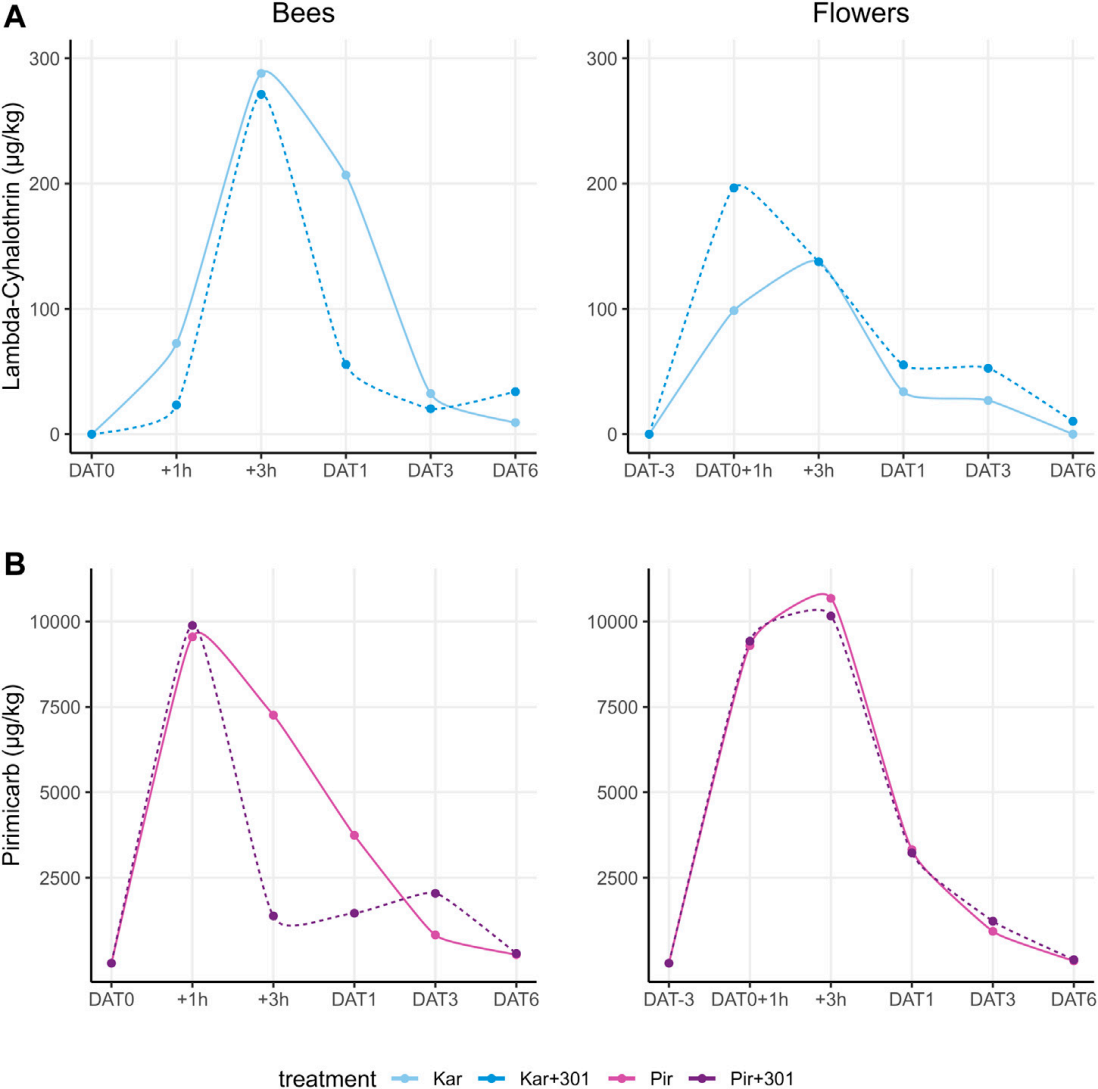
Measured residue levels for the active substances lambda-cyhalothrin and pirimicarb in the matrices dead bees (dead bee trap) **(A)** and flowers (blossoms and stems) **(B)** during the tunnel phase including pre- and post-exposure.

Lambda-cyhalothrin showed similar residue peaks in dead bees 3 h after application for both Karate Zeon treatments irrespective of the adjuvant: Kar: 279.0 μg/kg and Kar+301: 271.2 μg/kg (Figure 6A). However, at DAT1, Kar+301 (55.7 μg/kg) showed a 4-fold faster degradation compared to Kar without BreakThru S 301 (206.7 μg/kg). In flowers, the peak for Kar was reached 3 h after application, while Kar+301 peaked 1 h after application. In the latter, a similar range was measured as in dead bees (196.6 μg/kg). In contrast, Kar showed a 1.4-fold lower peak (137.6 μg/kg) compared to Kar+301.

Pirimicarb had similar residue peaks in dead bees 1 h after application for both Pirimor Granulat treatments, regardless of the use of the adjuvant: Pir: 9,550.4 μg/kg and Pir+301: 9,885.3 μg/kg (Figure 6B). 2 h later, on DAT+3h, a 5-fold decrease of pirimicarb in Pir+301 (1,381.6 μg/kg) compared to Pir (7,260.2 μg/kg) was detected. In flowers, the peak for pirimicarb was reached 3 h after application in both Pir (10,678.5 μg/kg) and Pir+301 (10,161.9 μg/kg). Here, both courses were nearly identical.

## 4 Discussion

Agricultural spray adjuvants are considered inert because they do not contain active substances (EPA, 2022). Therefore, instructions for use usually do not consider the exposure of bees and so may be applied directly to the flowering crop (BVL, 2023b). However, under laboratory conditions, we have already demonstrated that some of these adjuvants enhance the effects of various classes of insecticides, leading to higher mortality in honey bees (Wernecke et al., 2022). Yet, it remains unclear how these effects can be translated to honey bee colonies under free-flying conditions and field-realistic scenarios. To date, very few studies exist that have investigated the effects of adjuvants on different parameters of bee health. Much of the knowledge so far is based on laboratory studies, but there are only few studies under field conditions (Straw, et al., 2022). A more realistic but controlled approach to evaluate potential synergies between adjuvants and insecticides will increase knowledge of these compounds and could improve pollinator safety efforts.

For this reason, we assessed colony parameters in a worst-case spray scenario. In a semi-field approach, we applied two common insecticide formulations either alone or in a tank mixture combined with an OSS spray adjuvant to test for possible toxicity increasing effects on exposed bees and colonies.

We found that the OSS in combination with the applied insecticides had no significant adverse effects on bee mortality, colony or brood development, besides a reduced flower visitation for the pirimicarb tank mixture compared to the insecticide alone. Unlike Karate Zeon, both Pirimor Granulat treatments resulted in a significantly lower flower visitation compared to the control considering the whole exposure phase.

### 4.1 Mortality

In the semi-field study, mortality results are contrasting the findings obtained in our study under laboratory conditions (Wernecke et al., 2022). Here, Karate Zeon and Pirimor Granulat in combination with the OSS Break-Thru S 301 caused 100% and 63.8% mortality 72-h after contact exposure. The OSS adjuvant increased the toxicity of both insecticides significantly when compared to the insecticide alone (Wernecke et al., 2022). In contrast, the semi-field results suggest that the adjuvant did not significantly increase the toxicity of the two insecticides.

This absence of increased mortality at the next higher test level is a well-known phenomenon, which results from a variety of (environmental) factors that occur under realistic field conditions and the buffering capacity of the bee colonies (Alix and Lewis, 2010; Henry et al., 2015; Wernecke et al., 2019). Laboratory studies involve an artificially controlled environment in which honey bees are kept in small groups in experimental cages, contrary to their natural habitat, which is a limitation *per se* (EPPO, 2010). Here, a significantly higher stress level of the individual bee is achieved compared to the naturally environment in the colony, which negatively affects the pesticide tolerance of the bee (Harwood and Dolezal, 2020). An example of this is provided by De Smet et al. (2017) who exposed honey bees to imidacloprid and found general immunosuppression in laboratory experiments without detoxification mechanisms becoming active. In contrast, bees in the field showed stimulated expression of certain detoxification genes with robust immune responses (De Smet et al., 2017). This demonstrates that bees under semi-field conditions can counteract chemical stress differently than in the laboratory. In contrast to the controlled environment, in the field also temperature, weather, time of day, or, for example, the time interval between application and foraging, influence the actual exposure concentration of foragers (Kordecki, 2019). Additional to these factors, reduced exposure as a result of the three-dimensional structure of the crop or the dilution effect in the colony is possible. At the colony level, the fate of individual bees and minor effects can be masked due to the high buffering capacity of the superorganism (Henry et al., 2015). This means that pesticide exposure may cause damage to individual honey bees, but as long as colony functionality and reproduction is maintained, these losses can be buffered and compensated for within the superorganism (Odemer et al., 2020). All of these factors could be an explanation for this discrepancy.

### 4.2 Flower visitation

Contrary to the unaffected mortality, a repellent effect of the carbamate was observed as both Pirimor Granulat treatments resulted in a reduced flower visitation of foragers compared to all other groups. As carbamates were originally designed to repel insect pests, this is usually to be expected (Matteson and Taft, 1963; Padilla, 2005). However, in contrast to the evaluation of the individual time points, the overall evaluation of the exposure phase showed a significant reduction in flower visits in the case of the carbamate adjuvant tank mixture compared to the carbamate application alone. This effect could not be observed for the pyrethroid. Nevertheless, shortly after spray application (DAT0+1h), there was a considerable decrease in flower visitation in all insecticide treatments, whereas in the control group the number of foragers per plot increased compared to pre-application levels. As with carbamates, pyrethroids such as lambda-cyhalothrin are are also known to have repellent effects on bees (reviewed in Thompson, 2003). This in turn probably leads to a reduction in exposure, which lowers the lethal risk for foragers that were not directly oversprayed (reviewed in Thompson, 2001) and may contribute to the low mortality results compared to the laboratory (where direct exposure was forced).

### 4.3 Colony development

Another parameter considered in the study was the honey bee colony development reflected in the number of bees and brood cells. Here, the population data followed a similar trend in all groups and a typical development pattern under semi-field conditions. After the colonies were placed inside the tunnel, the existing pupae hatched, which initially increased the number of bees. The number of brood cells however, decreased in the tunnel. This effect is known as “caging effect” (Lückmann and Becker, 2015) and results from the removal of eggs and newly hatched larvae due to limited resources (Szczesniak et al., 2018). The start of the monitoring phase outside the tunnels counteracted this effect. On DAT18, an increasing trend in the number of brood cells and bees was again observed in all treatment groups.

Based on these results, neither an influence by the insecticides alone nor an increase in effects by the adjuvant-insecticide tank mixture on colony development is evident.

### 4.4 Brood development

Another particularly vulnerable life stage of bees is the brood development and negative effects could affect generations of offspring even at sublethal levels (Tomé et al., 2020). OSS have been reported to increase larval susceptibility to Black Queen Cell Virus in the laboratory (Fine et al., 2017) and are even capable of inducing brood mortality on their own (Kordecki, 2019). However, this has not been empirically verified under colony conditions. For that reason, we used a complete brood cycle to measure brood development.

Significant brood effects did not occur. However, in the treatments with the respective insecticide-adjuvant tank mixture a trend for elevated median brood termination rates was observed. The modified method of Schur et al. (2003), which was used for brood assessment, was found to overlook delayed development of bees due to pesticide exposure (Odemer et al., 2020). Hence, the raw data was carefully reviewed to identify brood cells that were still capped at the end of their development. Consistent with colony development (bee and brood cell numbers), yet brood termination rates did not confirm delayed hatching in any of the treatments.

However, our results regarding BTR should be interpreted with caution. Lückmann and Tänzler (2020) noted that BTR is less than 20% under field conditions, which we confirmed in a previous study (Odemer et al., 2020). In tunnels, a generally average control brood termination rate of ∼30% in studies conducted in Germany was recorded (Szczesniak et al., 2018). Our results provide slightly higher values, indicating suboptimal conditions for brood rearing, even though a flowering crop was present. Pistorius et al. (2012) further highlighted that the ratio of crop area to colony strength plays a crucial role for successful colony development under semi-field conditions. They suggest ideally using >80 m^2^ of crop area and a colony strength of about 7,000 bees per replicate. We used 10,000 bees’ strong colonies in plots of 40 m^2^, to which the observed high BTRs can most likely be attributed.

### 4.5 Residue analysis

Given that adjuvants are generally intended to improve leaf surface wetting or deposition, penetration, and uptake of PPPs, they can likewise influence final residue levels as well as the rate of PPP degradation (Xu et al., 2019). Especially pyrethroids are known for their UV susceptibility and rapid degradation of active substances. Adjuvants, such as adhesives, however, can increase the efficacy of pyrethroids by improving spray solution adhesion and forming a wax-like film on the plant surface that embeds the active substance (Schönberger et al., 2015). Due to the poor availability of data on residue impact, this study investigated whether and to what extent the adjuvant Break-Thru S 301, which is marketed to improve adhesion, wetting, and penetration (Alzchem, 2022), alters the residue behavior of both insecticides tested at semi-field conditions.

Our results could neither confirm nor completely exclude the assumption of changed fate profiles for the tested PPPs. The number of samples per test date was too low for a robust evaluation. The peak values were in a similar range both in the presence and absence of adjuvants. At best, the fate profile of the dead bees for both pirimicarb and lambda-cyhalothrin could indicate a tendency towards altered residue behavior due to the influence of adjuvants. However, this assumption must be corroborated by the collection of further data.

In addition, it is worth noting that there is very limited literature on the fate of adjuvants or their combination with pesticides in bee matrices or hive products. Straw et al. (2022) highlights that there are not more than three peer-reviewed studies on this topic as yet. They stress the importance of increasing knowledge and data on these substances since only residues from bee matrices allow realistic exposure scenarios to be considered in experiments. Adjuvants are released into the environment in large quantities (Mullin et al., 2015). Therefore, it is not surprising that residues can be detected in bee products such as beeswax and pollen (Chen and Mullin, 2013) or in groundwater (Krogh et al., 2002). Furthermore, a deeper understanding of adverse effects and their underlying mechanisms could improve the regulation of adjuvants and advance bee safety of plant protection products (Straw et al., 2022).

### 4.6 Limitations

A critical look at our study design reveals that our methodological approach did not include testing the single OSS and therefore we cannot directly compare results with studies that have done so. In fact, some authors consider OSS to be a standalone pesticide (Cowles et al., 2000) and have linked them to affecting health of entire honey bee populations (Mullin et al., 2016). However, because application of adjuvants alone is not common practice and no increased mortality was observed under laboratory conditions, we decided to omit testing of the single OSS. Yet, if there had been a strong effect of the adjuvant, this would have been reflected in at least one of the measured parameters in this study.

The use of tunnels for standardized conditions and exposure is legitimate and offers advantages (OECD, 2007). However, when considering colony conditions, it becomes evident that such a design also has limitations. Colony development showed a sharp decline in brood cells throughout the tunnel phase, which also affected BTR, as described under the point “Brood development”.

Even though OECD Guidance Document 75 recommends tunnels with a plot size of 40 m^2^ (OECD, 2007), our results suggest either drastically adjusting the colony size, increasing the plot size, or both to obtain reliable data. In our case, the reduction from 10,000 to 6,000 bees (as recommended in OECD, 2007) would have meant a strong intervention in the colony structure, which could have caused further problems. Nevertheless, the adaptation of the bee colonies to the conditions in the semi-field is very important and should only be performed at a level that the bees can cope with well.

## 5 Conclusion

It has recently been shown that OSS in particular can affect the health of honey bees. This evidence was mainly derived from laboratory studies. Our trial was therefore conducted under semi-field conditions with full-sized bee colonies and combinations of a commonly used OSS with a carbamate and a pyrethroid insecticide. In contrast to previous laboratory results, no negative effects on several important colony parameters, including population dynamics could be observed. This confirms once more that the effects found at laboratory level may not necessarily translate to the colony level. It is known that bee colonies buffer environmental stress through mechanisms that are not yet fully understood. Due to practical reasons one adjuvant in combination with two different insecticides were tested not being representative for all possible mixtures. However, the selection was based on a preliminary screening test in the laboratory to identify mixing partners likely to cause effects in higher tier tests. Hence, for a better understanding of mixing effects and a scientifically sound risk assessment, further work in this field is required. To safeguard bee pollinators in agricultural landscapes I) reliable methods should be developed to detect effects at the smallest biological relevant scale, II) a wider range of possible tank-mix partners to which pollinators are exposed should be considered, III) the fate of adjuvants in the environment should be monitored and their toxicity increasing potential further evaluated when mixed with pesticides.

## Data Availability

The raw data supporting the findings of our study are available at the OSF repository at https://osf.io/3f5a9/ or at DOI: https://10.17605/OSF.IO/3F5A9.
