# Supplementary material for: A selected organosilicone spray adjuvant does not enhance lethal effects of a pyrethroid and carbamate insecticide on honey bees
Source: Front Physiol. 2023 Jun 1;14:1171817. doi: 10.3389/fphys.2023.1171817 (PMC10267468; doi:10.3389/fphys.2023.1171817)
Supplement: Supplementary file 1 [file DataSheet1.docx]

**Organosilicone spray adjuvant does not enhance lethal effects of a pyrethroid and carbamate insecticide on honey bees under semi-field conditions**

**Anna Wernecke^1^, Jakob H. Eckert*^1,4^, Gabriela Bischoff^2^, Rolf Forster^3^, Jens Pistorius^1^, Richard Odemer^1^**

^1^Julius Kühn-Institut (JKI) – Federal Research Centre for Cultivated Plants, Institute for Bee Protection, Messeweg 11/12, 38104 Braunschweig, Germany

^2^Julius Kühn-Institut (JKI) – Federal Research Centre for Cultivated Plants, Institute for Bee Protection, Königin-Luise-Straße 19, 14195 Berlin, Germany

^3^Bundesamt für Verbraucherschutz und Lebensmittelsicherheit (BVL) – Federal Office of Consumer Protection and Food Safety, Bundesallee 51, 38116 Braunschweig, Germany

^4^Institute of Microbiology, Technische Universität Braunschweig, Spielmannstraße 7,
38106 Braunschweig, Germany

*** Correspondence:**Corresponding author: [jakob.eckert@julius-kuehn.de](mailto:jakob.eckert@julius-kuehn.de), Tel.: +49 (0) 3946 47 7238

ORCID:
Jakob H. Eckert 0000-0002-6678-2442
Richard Odemer 0000-0003-2230-4294
Anna Wernecke 0000-0003-1264-1828

# SUPPLEMENTARY MATERIAL

**S1 Brood development and photographic assessment**

# **Supplementary Method S1:** Brood development and photographic assessment adopted from OECD 2007 and based on Schur et al. (2003).

**Brood termination rate (BTR)**

Based on the brood termination rate the failure of individual eggs or larvae to develop is quantitatively assessed. For the calculation of the brood termination rate the observed cells are split into two categories:

- The bee brood in the observed cell reached the expected brood stage at the different assessment days or was found empty or containing an egg after hatch of the adult bee on BFD+21 → **successful development**

Example:

ID BFD0 BFD+6 BFD+11 BFD+17 BFD+21

- The bee brood in the observed cell did not reach the expected brood stage at one of the assessment days or food was stored in the cell during BFD +6 to +17 → **termination of the bee brood development**

Example:

ID BFD0 BFD+6 BFD+11 BFD+17 BFD+21

For the final calculation the number of cells, where termination of the bee brood development was recorded, is summed up for each treatment and colony, is multiplied by 100 and divided by the number of cells observed to obtain the brood termination rate in %.

**Table S1.1:** Brood area Fixing Day (BFD) assessments during the course of the study, modified after Schur et al. (2003). The numbers for the brood index were assigned to the cell content, respectively. If the expected brood stage was met at the specified BFD, the cell was labeled “1”, if not as terminated (Brood termination = 0). To differentiate brood stages in the photographic assessment, a color code was used automatically by the HiveAnalyzer software.


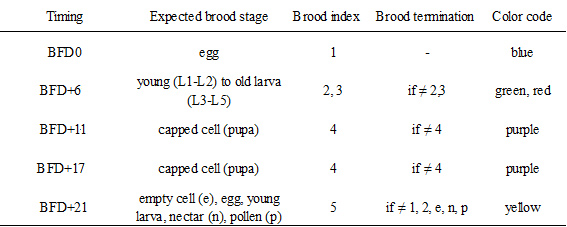


**Supplementary Table S1.2:** Brood Development data (BFD)

**Table S1.2:** Brood termination rates 18 days after application (BFD+21) for the five treatments (Control, Karate Zeon, Karate Zeon + Break-Thru S 301, Pirimor Granulat, Pirimor Granulat + Break-Thru S 301).

|  | *Control (water)* | | |  | *Karate Zeon* | | |  | *Karate Zeon + Break-Thru S 301* | | |  | *Pirimor Granulat* | | |  | *Pirimor Granulat + Break-Thru S 301* | | |
| --- | --- | --- | --- | --- | --- | --- | --- | --- | --- | --- | --- | --- | --- | --- | --- | --- | --- | --- | --- |
|  | *total number of cells (BFD0)* | *brood termination (cell count BFD+21)* | *BTR [%]* |  | *total number of cells (BFD0)* | *brood termination (cell count BFD+21)* | *BTR [%]* |  | *total number of cells (BFD0)* | *brood termination (cell count BFD+21)* | *BTR [%]* |  | *total number of cells (BFD0)* | *brood termination (cell count BFD+21)* | *BTR [%]* |  | *total number of cells (BFD0)* | *brood termination (cell count BFD+21)* | *BTR [%]* |
| colony 1A | 300 | 22 | 7.3 |  | 300 | 66 | 22.0 |  | 300 | 96 | 32.0 |  | - | - | - |  | 300 | 75 | 25.0 |
| colony 1B | 300 | 22 | 7.3 |  | 300 | 100 | 33.3 |  | 300 | 187 | 62.3 |  | 300 | 92 | 30.7 |  | 300 | 129 | 43.0 |
| **colony 1** | **600** | **44** | **7.3** |  | **600** | **166** | **27.7** |  | **600** | **283** | **47.2** |  | **300** | **92** | **30.7** |  | **600** | **204** | **34.0** |
| colony 2A | 300 | 173 | 57.7 |  | 300 | 67 | 22.3 |  | 300 | 96 | 32.0 |  | 300 | 123 | 41.0 |  | 300 | 263 | 87.7 |
| colony 2B | 300 | 197 | 65.7 |  | 300 | 106 | 35.3 |  | 300 | 105 | 35.0 |  | 300 | 116 | 38.7 |  | 300 | 285 | 95.0 |
| **colony 2** | **600** | **370** | **61.7** |  | **600** | **173** | **28.8** |  | **600** | **201** | **33.5** |  | **600** | **239** | **39.8** |  | **600** | **548** | **91.3** |
| colony 3A | 300 | 103 | 34.3 |  | 300 | 138 | 46.0 |  | 300 | 96 | 32.0 |  | 300 | 88 | 29.3 |  | 300 | 94 | 31.3 |
| colony 3B | 300 | 177 | 59.0 |  | 300 | 113 | 37.7 |  | 300 | 123 | 41.0 |  | 300 | 111 | 37.0 |  | 300 | 51 | 17.0 |
| **colony 3** | **600** | **280** | **46.7** |  | **600** | **251** | **41.8** |  | **600** | **219** | **36.5** |  | **600** | **199** | **33.2** |  | **600** | **145** | **24.2** |
| colony 4A | 157 | 59 | 37.6 |  | 300 | 32 | 10.7 |  | 300 | 244 | 81.3 |  | 300 | 126 | 42.0 |  | 289 | 207 | 71.6 |
| colony 4B | 300 | 122 | 40.7 |  | 300 | 73 | 24.3 |  | 300 | 197 | 65.7 |  | 300 | 127 | 42.3 |  | 300 | 193 | 64.3 |
| **colony 4** | **457** | **181** | **39.6** |  | **600** | **105** | **17.5** |  | **600** | **441** | **73.5** |  | **600** | **253** | **42.2** |  | **589** | **400** | **67.9** |
|  |  |  |  |  |  |  |  |  |  |  |  |  |  |  |  |  |  |  |  |
| Mean |  |  | 38.8 |  |  |  | 29.0 |  |  |  | 47.7 |  |  |  | 36.5 |  |  |  | 54.4 |
| STABW |  |  | 22.9 |  |  |  | 10.0 |  |  |  | 18.2 |  |  |  | 5.4 |  |  |  | 31.0 |
| SE |  |  | 11.5 |  |  |  | 5.0 |  |  |  | 9.1 |  |  |  | 2.7 |  |  |  | 15.5 |

BFD = Brood fixing day; BTR = brood termination rate
A and B indicate broodcomb side A and broodcomb side B

**S2 Residue analysis**

# **Supplementary Method S2:** Analytical method verification.

The analytical method was verified with the sample materials bees and oilseed rape. The untreated rapeseed plants were used for the preparation of matrix standards. The results of the method verification for the target substances are summarized in **Tables S2.1** and **S2.2**.

**Table S2.1:** Quality parameters REC, LOD, LOQ used to evaluate the results for oilseed rape.

| ***oilseed rape (flowers and stems)*** | *20 µg/kg (n=5)* | | |  | |
| --- | --- | --- | --- | --- | --- |
| *active ingredients* | *REC*  *[%]* | *ASD*  *[%]* | *RSD*  *[%]* | *LOD*  *[µg/kg]* | *LOQ*  *[µg/kg]* |
| lambda-cyhalothrin | 67 | 6 | 9 | 0.40 | 0.80 |
| pirimicarb | 98 | 2 | 2 | 0.02 | 0.04 |
| pirimicarb-desmethyl | 97 | 4 | 4 | 0.04 | 0.08 |
| pirimicarb-desmethylformamido | 104 | 3 | 3 | 0.20 | 0.40 |

REC = recovery; ASD/RSD = absolute / relative standard deviation; LOD = limit of detection; limit of quantification

**Table S2.2:** Quality parameters REC, LOD, LOQ used to evaluate the results for dead bees.

| ***dead bees*** | *20 µg/kg (n=5)* | | |  | |
| --- | --- | --- | --- | --- | --- |
| *active ingredients* | *REC*  *[%]* | *ASD*  *[%]* | *RSD*  *[%]* | *LOD*  *[µg/kg]* | *LOQ*  *[µg/kg]* |
| lambda-cyhalothrin | 84 | 3 | 4 | 2.0 | 4.0 |
| pirimicarb | 102 | 2 | 2 | 0.10 | 0.20 |
| pirimicarb-desmethyl | 98 | 4 | 4 | 0.04 | 0.10 |
| pirimicarb-desmethylformamido | 102 | 3 | 3 | 1.0 | 2.0 |

REC = recovery; ASD/RSD = absolute / relative standard deviation; LOD = limit of detection; limit of quantification

The calculations for LOD and LOQ are based on the parameters of the preparation of the project samples. The following data were used here as examples: for rapeseed: 5 g in 2 ml measuring solution, for bees: 1 g in 2 ml measuring solution.

The determination of the LOD and LOQ values was based on the measurement results for the matrix standards, which were also measured in the sample series. The LOD was defined as the lowest concentration at which at least two MRMs or SRMs were detected whose signals were at least three times higher than the background noise of the chromatogram and whose ratio was within the range of the required criteria (SANTE, 2020).

The LOQ, according to the requirements of the above SANTE guideline, is the lowest additional concentration of validation at which mean recoveries in the range of 70-120% with RSD of
≤ 20% are obtained for all analytes after application of the full method. Recoveries outside the range of 70-120% are acceptable if consistent (RSD ≤ 20%), but the mean recovery should not be lower than 30% or greater than 140%. These requirements are mostly fulfilled by the validation data obtained (**Tables S2.1** and **S2.2**).

The recovery rate of lambda-cyhalothrin is below 70% (with RSD of 9%) in the additional experiment with oilseed rape. This is probably due to the low water solubility of the active ingredient in combination with the respective sample material and the conditions of the multi-method.

Since it was not possible to perform a large number of validation experiments to determine the LOQ, the next higher concentration of the calibration standards above the detection limit was set as the LOQ. The signals of the MRMs selected for the target analytes were at least 10 times higher than the background noise of the chromatogram in the matrix standards set as LOQ.

**Supplementary Table S2.3:** Residue values of dead bees

**Table S2.3:** Residue values of dead bees (a.s. in µg/kg) for sampling times before and after application for the five treatments (Control, Karate Zeon, Karate Zeon + Break-Thru S 301, Pirimor Granulat, Pirimor Granulat + Break-Thru S 301).

|  | *Control (water)* | | | |  | *Karate Zeon* |  | *Kar+301* |  | *Pirimor Granulat* | | |  | *Pirimor Granulat + Break-Thru S 301* | | |
| --- | --- | --- | --- | --- | --- | --- | --- | --- | --- | --- | --- | --- | --- | --- | --- | --- |
|  | *lambda- cyhalothrin* | *pirimicarb* | *pirimicarb-desmethyl* | *pirimicarb-desmethyl-formamido* |  | *lambda- cyhalothrin* |  | *lambda- cyhalothrin* |  | *pirimicarb* | *pirimicarb-desmethyl* | *pirimicarb-desmethyl-formamido* |  | *pirimicarb* | *pirimicarb-desmethyl* | *pirimicarb-desmethyl-formamido* |
| -0d | n.n. | n.n. | n.n. | n.n. |  | n.n. |  | n.n. |  | n.n. | n.n. | n.n. |  | n.n. | n.n. | n.n. |
| +1h | n.n. | n.n. | n.n. | n.n. |  | 72.56 |  | 23.34 |  | 9550.38 | 1975.45 | 42.62 |  | 9885.26 | 3551.45 | 34.57 |
| +3h | n.n. | n.n. | n.n. | n.n. |  | 287.99 |  | 271.23 |  | 7260.16 | 2164.84 | 22.71 |  | 1384.57 | 402.80 | 3.92 |
| +1d | n.n. | n.n. | n.n. | n.n. |  | 206.74 |  | 55.69 |  | 3744.09 | 2665.15 | 24.11 |  | 1436.54 | 732.11 | 26.23 |
| +3d | n.n. | n.n. | n.n. | n.n. |  | 32.47 |  | 20.32 |  | 827.77 | 468.68 | 13.61 |  | 2045.70 | 1213.37 | 21.62 |
| +6d | n.n. | n.n. | n.n. | n.n. |  | 9.29 |  | 33.92 |  | 251.14 | 193.96 | 24.82 |  | 283.38 | 174.42 | 8.83 |
| Kar+301= Karate Zeon + Break-Thru S 301 | | | | | | |  |  |  |  |  |  |  |  |  |  |

**Supplementary Table S2.4:** Residue values of plant material

**Table S2.4:** Residue levels of plant material (flowers and stems) (a.s. in µg/kg) for sampling times before and after application for the five treatments (Control, Karate Zeon, Karate Zeon + Break-Thru S 301, Pirimor Granulat, Pirimor Granulat + Break-Thru S 301).

|  | *Control (water)* | | | |  | *Karate Zeon* |  | *Kar+301* |  | *Pirimor Granulat* | | |  | *Pirimor Granulat + Break-Thru S 301* | | |
| --- | --- | --- | --- | --- | --- | --- | --- | --- | --- | --- | --- | --- | --- | --- | --- | --- |
|  | *lambda- cyhalothrin* | *pirimicarb* | *pirimicarb-desmethyl* | *pirimicarb-desmethyl-formamido* |  | *lambda- cyhalothrin* |  | *lambda- cyhalothrin* |  | *pirimicarb* | *pirimicarb-desmethyl* | *pirimicarb-desmethyl-formamido* |  | *pirimicarb* | *pirimicarb-desmethyl* | *pirimicarb-desmethyl-formamido* |
| -3d | n.n. | n.n. | n.n. | n.n. |  | n.n. |  | n.n. |  | n.n. | n.n. | n.n. |  | 0.03 | 0.06 | n.n. |
| +1h | n.n. | n.n. | n.n. | n.n. |  | 98.63 |  | 196.56 |  | 9269.86 | 1526.18 | 266.41 |  | 9425.70 | 1570.46 | 183.82 |
| +3h | n.n. | 0.49 | n.n. | n.n. |  | 137.60 |  | 137.54 |  | 10678.51 | 1772.31 | 325.32 |  | 10161.90 | 1423.05 | 227.22 |
| +1d | n.n. | 0.10 | n.n. | n.n. |  | 33.87 |  | 55.30 |  | 3326.02 | 1467.77 | 131.23 |  | 3232.09 | 1376.95 | 96.31 |
| +3d | n.n. | 0.37 | n.n. | n.n. |  | 26.93 |  | 52.63 |  | 933.93 | 881.38 | 61.12 |  | 1230.34 | 1094.04 | 73.88 |
| +6d | n.n. | n.n. | n.n. | n.n. |  | n.n. |  | 10.29 |  | 66.66 | 123.25 | 8.60 |  | 102.20 | 178.78 | 9.23 |
| Kar+301= Karate Zeon + Break-Thru S 301 | | | | | | |  |  |  |  |  |  |  |  |  |  |

**S3 Mortality & flower visitation**

# **Supplementary Results S3:** Model output mortality & flower visitation.


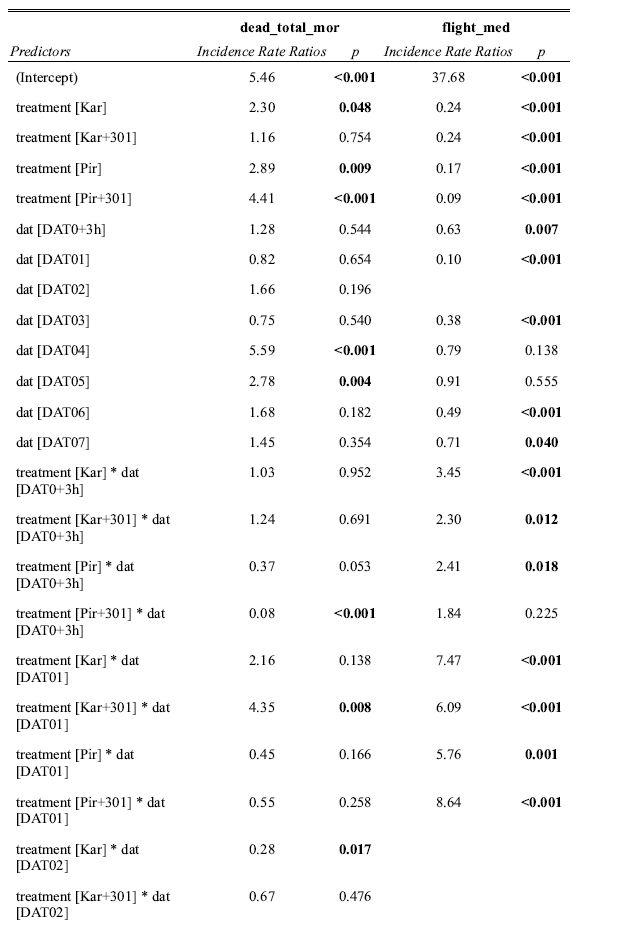


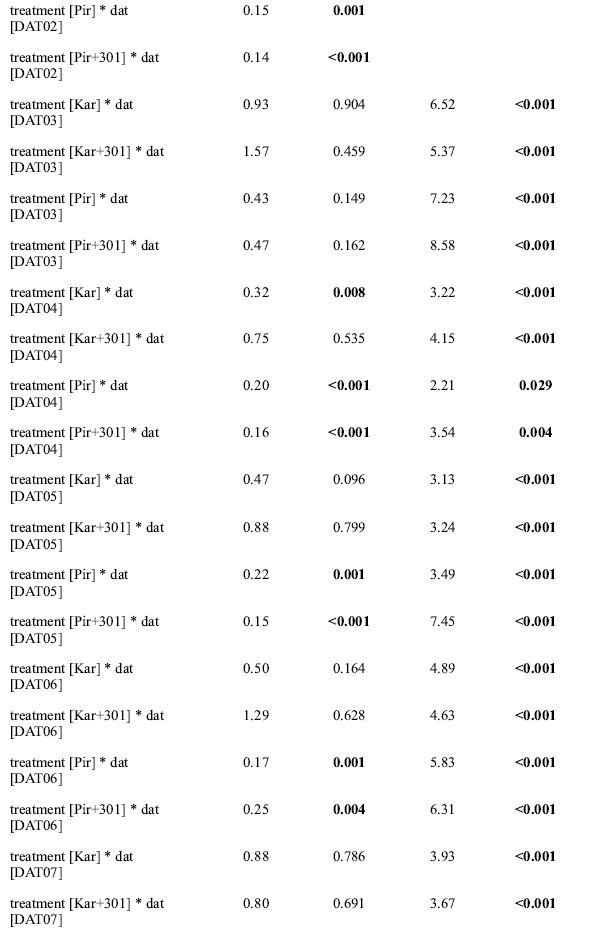


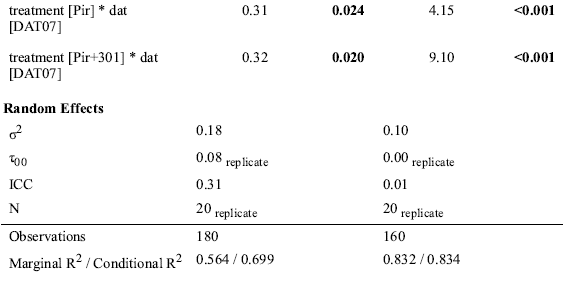


# **Supplementary Figure S3:** Mean number of dead + moribund bees and foragers per group.


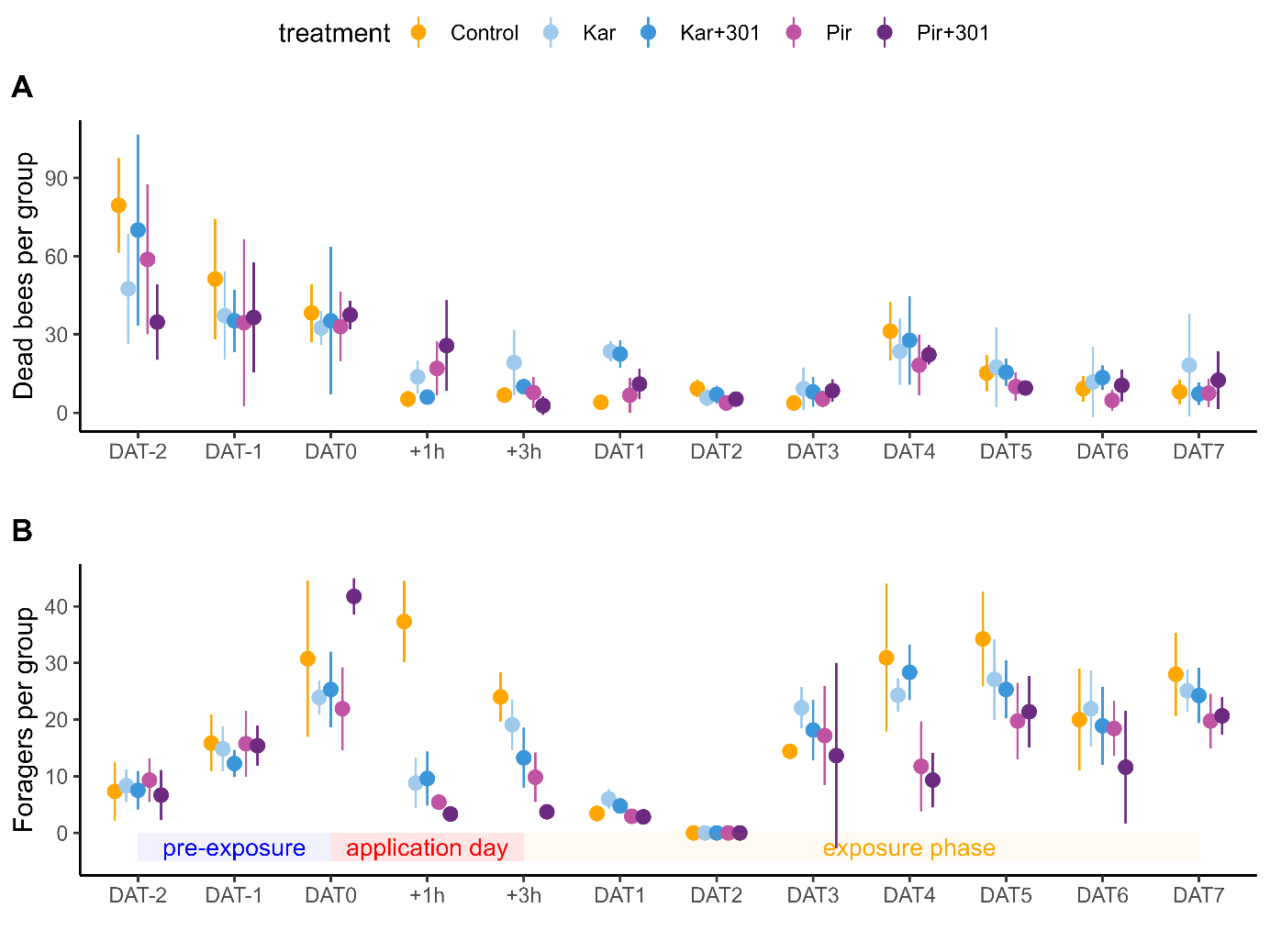


**Fig. S3:** Mean number (± SD) of dead worker bees (+ moribund bees) in the trap per colony (**A**) and foragers per quadrat and minute (**B**) in the semi-field trial for pre-exposure phase (DAT-2 to DAT0), application day (DAT0 to DAT0+3h) and exposure phase (DAT0+1h to DAT7). Treatments are defined as follows: Control (water), Kar (Karate Zeon), Kar+301 (Karate Zeon + Break-Thru S 301), Pir (Pirimor Granulat) and Pir+301 (Pirimor Granulat + Break-Thru S 301) (n_colonies_ = 4).

**Supplementary Table S3.1:** Mortality data.

**Table S3.1:** Number of dead and moribund worker bees in the death trap per colony (n=4/treatment) and time of recording for the five treatments (Control, Karate Zeon, Karate Zeon + Break-Thru S 301, Pirimor Granulat, Pirimor Granulat + Break-Thru S 301).

| *Day after* | *Control (water)* | | | |  | *Karate Zeon* | | | |  | *Karate Zeon + Break-Thru S 301* | | | |  | *Pirimor Granulat* | | | |  | *Pirimor Granulat + Break-Thru S 301* | | | |
| --- | --- | --- | --- | --- | --- | --- | --- | --- | --- | --- | --- | --- | --- | --- | --- | --- | --- | --- | --- | --- | --- | --- | --- | --- |
| treatment | *1* | *2* | *3* | *4* |  | *1* | *2* | *3* | *4* |  | *1* | *2* | *3* | *4* |  | *1* | *2* | *3* | *4* |  | *1* | *2* | *3* | *4* |
| DAT-2 | 59 | 73 | 30 | 80 |  | 64 | 21 | 65 | 40 |  | 46 | 111 | 33 | 90 |  | 92 | 73 | 31 | 39 |  | 41 | 52 | 20 | 26 |
| DAT-1 | 62 | 38 | 78 | 27 |  | 29 | 18 | 46 | 56 |  | 32 | 42 | 20 | 47 |  | 39 | 78 | 7 | 14 |  | 41 | 64 | 16 | 25 |
| DAT0-1h | 24 | 36 | 50 | 43 |  | 33 | 25 | 31 | 41 |  | 22 | 27 | 15 | 77 |  | 36 | 37 | 14 | 45 |  | 39 | 44 | 31 | 36 |
| DAT0+1h | 2 | 8 | 3 | 8 |  | 21 | 10 | 7 | 17 |  | 4 | 5 | 6 | 9 |  | 22 | 4 | 28 | 14 |  | 50 | 20 | 9 | 24 |
| DAT0+3h | 6 | 4 | 7 | 10 |  | 23 | 12 | 7 | 35 |  | 6 | 10 | 12 | 12 |  | 5 | 16 | 2 | 8 |  | 2 | 1 | 0 | 8 |
| DAT1 | 3 | 5 | 3 | 5 |  | 23 | 22 | 29 | 20 |  | 19 | 27 | 17 | 27 |  | 3 | 15 | 0 | 9 |  | 17 | 6 | 6 | 15 |
| DAT2 | 12 | 5 | 12 | 8 |  | 3 | 9 | 3 | 8 |  | 12 | 5 | 5 | 6 |  | 3 | 7 | 4 | 1 |  | 6 | 5 | 6 | 4 |
| DAT3 | 4 | 2 | 4 | 5 |  | 3 | 21 | 8 | 5 |  | 10 | 15 | 5 | 2 |  | 10 | 3 | 4 | 4 |  | 7 | 12 | 3 | 12 |
| DAT4 | 22 | 46 | 23 | 34 |  | 14 | 41 | 14 | 25 |  | 21 | 34 | 9 | 49 |  | 22 | 33 | 8 | 10 |  | 26 | 25 | 19 | 19 |
| DAT5 | 7 | 16 | 14 | 24 |  | 6 | 40 | 12 | 12 |  | 16 | 22 | 9 | 15 |  | 18 | 8 | 6 | 8 |  | 12 | 8 | 7 | 11 |
| DAT6 | 2 | 13 | 11 | 11 |  | 4 | 32 | 6 | 5 |  | 12 | 13 | 9 | 20 |  | 5 | 10 | 4 | 0 |  | 6 | 11 | 6 | 19 |
| DAT7 | 2 | 7 | 13 | 10 |  | 9 | 47 | 14 | 3 |  | 8 | 4 | 4 | 13 |  | 15 | 8 | 4 | 3 |  | 5 | 13 | 4 | 28 |

Dead and moribund worker bees were assessed and summarized as one count.

**Supplementary Table S3.2:** Flower visitation data.

**Table S3.2:** Average number of foraging bees per colony (n=4/treatment) and time of recording for the five treatments (Control, Karate Zeon, Karate Zeon + Break-Thru S 301, Pirimor Granulat, Pirimor Granulat + Break-Thru S 301).

| *Day after* | *Control (water)* | | | |  | *Karate Zeon* | | | |  | *Karate Zeon + Break-Thru S 301* | | | |  | *Pirimor Granulat* | | | |  | *Pirimor Granulat + Break-Thru S 301* | | | |
| --- | --- | --- | --- | --- | --- | --- | --- | --- | --- | --- | --- | --- | --- | --- | --- | --- | --- | --- | --- | --- | --- | --- | --- | --- |
| *treatment* | *1* | *2* | *3* | *4* |  | *1* | *2* | *3* | *4* |  | *1* | *2* | *3* | *4* |  | *1* | *2* | *3* | *4* |  | *1* | *2* | *3* | *4* |
| DAT-2 | 13,0 | 3,7 | 10,3 | 2,3 |  | 8,0 | 8,3 | 5,0 | 12,0 |  | 3,7 | 5,7 | 11,0 | 9,7 |  | 7,7 | 15,0 | 6,3 | 8,3 |  | 4,7 | 13,3 | 4,0 | 4,7 |
| DAT-1 | 22.0 | 16.0 | 15.7 | 9.7 |  | 14.0 | 20.0 | 15.0 | 10.3 |  | 10.3 | 12.0 | 15.7 | 11.0 |  | 13.7 | 24.0 | 10.3 | 15.0 |  | 18.3 | 17.0 | 10.3 | 16.0 |
| DAT0-1h | 20.3 | 45.3 | 39.7 | 17.7 |  | 21.0 | 27.0 | 26.0 | 21.7 |  | 32.0 | 25.0 | 28.0 | 16.3 |  | 14.7 | 30.7 | 17.3 | 25.0 |  | 45.0 | 44.0 | 38.7 | 39.3 |
| DAT0+1h | 33.3 | 43.7 | 43.0 | 29.3 |  | 5.3 | 7.3 | 7.3 | 15.3 |  | 6.3 | 4.7 | 13.7 | 13.7 |  | 5.3 | 7.0 | 4.7 | 4.7 |  | 2.0 | 4.3 | 4.0 | 3.0 |
| DAT0+3h | 26.0 | 22.0 | 29.0 | 19.0 |  | 16.0 | 18.0 | 16.7 | 25.7 |  | 9.7 | 7.7 | 17.3 | 18.3 |  | 6.7 | 8.0 | 8.3 | 16.3 |  | 2.3 | 4.3 | 4.3 | 4.0 |
| DAT1 | 1.3 | 4.0 | 4.7 | 3.7 |  | 4.7 | 4.7 | 6.3 | 8.3 |  | 3.0 | 5.0 | 6.3 | 4.7 |  | 2.3 | 3.7 | 3.7 | 2.0 |  | 1.3 | 3.0 | 3.3 | 3.7 |
| DAT2 | 0.0 | 0.0 | 0.0 | 0.0 |  | 0.0 | 0.0 | 0.0 | 0.0 |  | 0.0 | 0.0 | 0.0 | 0.0 |  | 0.0 | 0.0 | 0.0 | 0.0 |  | 0.0 | 0.0 | 0.0 | 0.0 |
| DAT3 | 14.0 | 14.0 | 15.3 | 14.3 |  | 17.0 | 22.0 | 24.7 | 24.7 |  | 12.3 | 15.3 | 20.7 | 24.3 |  | 5.7 | 20.0 | 16.3 | 26.7 |  | 8.3 | 5.3 | 3.0 | 38.0 |
| DAT4 | 49.7 | 29.7 | 24.3 | 20.0 |  | 27.0 | 26.7 | 21.3 | 22.3 |  | 25.7 | 26.0 | 35.7 | 26.0 |  | 14.3 | 21.3 | 8.7 | 2.7 |  | 8.3 | 12.0 | 14.0 | 3.0 |
| DAT5 | 29.0 | 44.3 | 37.7 | 26.0 |  | 29.0 | 28.7 | 33.7 | 17.0 |  | 22.3 | 29.3 | 30.0 | 19.7 |  | 17.3 | 17.3 | 29.7 | 14.7 |  | 16.0 | 18.0 | 30.3 | 21.3 |
| DAT6 | 33.3 | 17.7 | 15.0 | 14.0 |  | 21.0 | 31.7 | 17.3 | 17.7 |  | 28.0 | 11.3 | 17.3 | 19.0 |  | 22.3 | 21.7 | 11.7 | 18.0 |  | 24.7 | 3.7 | 4.0 | 14.0 |
| DAT7 | 31.3 | 31.0 | 32.7 | 17.0 |  | 21.0 | 30.0 | 24.7 | 24.7 |  | 29.7 | 20.0 | 27.0 | 20.3 |  | 19.0 | 19.7 | 14.3 | 26.0 |  | 21.3 | 21.7 | 23.7 | 16.0 |

Flower visitation based on three estimates per tent
rain 2 days after application

# **S4 Colony development**

# **Supplementary Results S4:** Model output colony development (bees & brood).


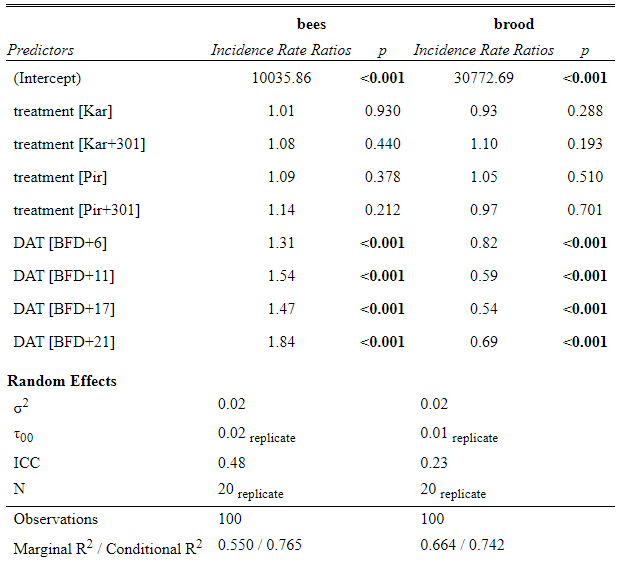


**Supplementary Table S4.1:** Colony development data – bees (estimation according to Liebefeld)

**Table S4.1:** Estimated number of bees per colony (n=4/treatment) and time of recording for the five treatments (Control, Karate Zeon, Karate Zeon + Break-Thru S 301, Pirimor Granulat, Pirimor Granulat + Break-Thru S 301).

| Day after | *Control (water)* | | | |  | *Karate Zeon* | | | |  | *Karate Zeon + Break-Thru S 301* | | | |  | *Pirimor Granulat* | | | |  | *Pirimor Granulat + Break-Thru S 301* | | | |
| --- | --- | --- | --- | --- | --- | --- | --- | --- | --- | --- | --- | --- | --- | --- | --- | --- | --- | --- | --- | --- | --- | --- | --- | --- |
| treatment | *1* | *2* | *3* | *4* |  | *1* | *2* | *3* | *4* |  | *1* | *2* | *3* | *4* |  | *1* | *2* | *3* | *4* |  | *1* | *2* | *3* | *4* |
| DAT-3 | 9490 | 10920 | 11765 | 10075 |  | 13520 | 7670 | 10985 | 11115 |  | 10335 | 9165 | 13520 | 12610 |  | 10270 | 10920 | 9880 | 11180 |  | 10400 | 11310 | 10400 | 8905 |
| DAT3 | 11440 | 14950 | 15080 | 12935 |  | 14495 | 11570 | 14105 | 15015 |  | 13065 | 14040 | 15860 | 16055 |  | 15600 | 13455 | 12805 | 14690 |  | 12350 | 14950 | 14625 | 14235 |
| DAT8 | 16445 | 16510 | 17745 | 14820 |  | 16900 | 12025 | 16705 | 19175 |  | 13910 | 17095 | 19370 | 18590 |  | 17810 | 14885 | 13585 | 16640 |  | 14105 | 18200 | 18850 | 17160 |
| DAT14 | 11245 | 13585 | 18005 | 17875 |  | 16835 | 10335 | 14950 | 20215 |  | 11310 | 13325 | 16380 | 21385 |  | 15795 | 13520 | 16380 | 18720 |  | 12415 | 21515 | 18395 | 14820 |
| DAT18 | 10400 | 15405 | 17615 | 24440 |  | 17615 | 12610 | 17810 | 18850 |  | 13390 | 16965 | 20280 | 27820 |  | 22815 | 19370 | 19500 | 27495 |  | 13260 | 31005 | 24310 | 31200 |

**Supplementary Table S4.2:** Colony development data – brood cells (estimation according to Liebefeld)

**Table S4.2:** Estimated number of brood cells (sum of eggs, larvae, pupae) per colony (n=4/treatment) and time of recording for the five treatments (Control, Karate Zeon, Karate Zeon + Break-Thru S 301, Pirimor Granulat, Pirimor Granulat + Break-Thru S 301).

| Day after | *Control (water)* | | | |  | *Karate Zeon* | | | |  | *Karate Zeon + Break-Thru S 301* | | | |  | *Pirimor Granulat* | | | |  | *Pirimor Granulat + Break-Thru S 301* | | | |
| --- | --- | --- | --- | --- | --- | --- | --- | --- | --- | --- | --- | --- | --- | --- | --- | --- | --- | --- | --- | --- | --- | --- | --- | --- |
| treatment | *1* | *2* | *3* | *4* |  | *1* | *2* | *3* | *4* |  | *1* | *2* | *3* | *4* |  | *1* | *2* | *3* | *4* |  | *1* | *2* | *3* | *4* |
| DAT-3 | 34800 | 31800 | 33400 | 21600 |  | 30400 | 24800 | 30800 | 31600 |  | 29800 | 33000 | 37400 | 35400 |  | 30000 | 30000 | 31400 | 31000 |  | 28800 | 36600 | 31200 | 23200 |
| DAT3 | 25400 | 25600 | 30400 | 19200 |  | 20000 | 23200 | 19800 | 30600 |  | 20000 | 21400 | 28000 | 33600 |  | 23000 | 21400 | 30200 | 30000 |  | 23400 | 31000 | 25000 | 25400 |
| DAT8 | 16600 | 24600 | 16400 | 16400 |  | 15800 | 13800 | 18400 | 16600 |  | 15400 | 21800 | 14600 | 29800 |  | 20000 | 13600 | 24600 | 20000 |  | 16600 | 21800 | 19800 | 13800 |
| DAT14 | 15800 | 17400 | 16200 | 16800 |  | 16000 | 14200 | 19600 | 15600 |  | 17400 | 15200 | 13400 | 23800 |  | 16600 | 13600 | 16600 | 25800 |  | 14800 | 12400 | 17400 | 19200 |
| DAT18 | 20000 | 24000 | 20400 | 20200 |  | 12200 | 18800 | 18000 | 18400 |  | 29000 | 20600 | 26000 | 29000 |  | 27800 | 17000 | 22400 | 25200 |  | 19800 | 20000 | 17400 | 18000 |

**References**

SANTE/12682/2019 (implemented 01/01/2020). Method Validation and Quality Control Procedures for Pesticide Residues Analysis in Food and Feed, EU Reference Laboratories for Residues of Pesticides.

Schur, A., Tornier, I., Brasse, D., Mühlen, W., Von Der Ohe, W., Wallner, K., and Wehling, M. (2003). Honey bee brood ring-test in 2002: method for the assessment of side effects of plant protection products on the honey bee brood under semi-field conditions. Bulletin of Insectology 56:91–96.
